# Prevalence of Congenital Aortic Valve Regurgitation in a Newborn Cohort

**DOI:** 10.1007/s00246-025-04149-0

**Published:** 2026-01-20

**Authors:** Aysha F. Farooqui, Jakob B. Norsk, Anna A. Raja, Vibeke Hjortdal, Julian K. Bjerrekaer, Marie Reindahl Fagerlund, R. Ottilia Vøgg, Christian Pihl, Henning Bundgaard, Kasper Iversen, Anne-Sophie Sillesen

**Affiliations:** 1 Department of Cardiology, Herlev-Gentofte University Hospital, Copenhagen, Denmark; 2 Department of Cardiology, The Heart Center, University Hospital Rigshospitalet, Copenhagen, 2100 Denmark; 3 Department of Thoracic Surgery, University Hospital Rigshospitalet, Copenhagen, Denmark; 4 Statens Serum Institut, Copenhagen, Denmark; 5 Department of Clinical Medicine, University of Copenhagen, Copenhagen, Denmark

**Keywords:** Aortic insufficiency, Echocardiography, Neonate, Aortopathy, Population study

## Abstract

Congenital aortic valve regurgitation is seen in approximately 1 in 4,000 live-births globally. This study investigated the prevalence and early impacts of aortic regurgitation in a newborn cohort. Between April 2016 and October 2018, all newborns in the Copenhagen Area were offered systematic transthoracic echocardiography in a prospective, multicenter population-based study. Aortic valve function was categorized as no, trivial (glimpse of regurgitant flow and/or vena contracta < 0.1 cm), or non-trivial aortic regurgitation (vena contracta ≥ 0.1 cm). Newborns with non-trivial regurgitation were matched 1:4 with controls (no regurgitation). Among 25,590 newborns, aortic regurgitation was detected in 329 newborns (52% male, median age 11 days), i.e. a prevalence of 1.3% (95% CI, 1.15%-1.43%), with 260 (79%) classified as trivial and 69 (21%) as non-trivial. Bicuspid aortic valve was more common in newborns with non-trivial regurgitation (13.0%) than controls (1.1%) (*p* < 0.001). Newborns with non-trivial regurgitation had larger aortic dimensions compared to controls (mean ± SD): aortic valve annulus (7.3 ± 0.7 mm vs. 7.0 ± 0.6 mm), Sinuses of Valsalva (10.3 ± 1.0 mm vs. 9.7 ± 1.0 mm), sino-tubular junction (8.4 ± 0.8 mm vs. 8.0 ± 0.8 mm), and ascending aorta diameter (10.0 ± 0.9 mm vs. 9.4 ± 1.1 mm) (all *p* < 0.006). Left ventricular dimensions and function did not differ between groups. Congenital aortic regurgitation was present in 1.3% of all newborns. Non-trivial regurgitation (0.3%) was associated with larger aortic dimensions and higher prevalence of bicuspid aortic valve (13%), but no early signs of left ventricular impairment. Follow-up will help identify those at risk of progression, potentially due to valvular and/or aortic pathology, who may benefit from regular surveillance, and those who can be safely discontinued.

*Trial registration* ClinicalTrials.gov NCT02753348 (registered April 27th 2016).

## Introduction

Congenital heart disease is the most common birth defect, estimated to affect 8.0 to 9.4 per 1,000 live births [1, 2]. The global prevalence of aortic regurgitation is approximately 1 in every 4,000 live-births [2]. Aortic regurgitation in newborns and children in Western Europe is primarily due to congenital malformations of the aortic valve, particularly bicuspid aortic valve [3]. It may also be seen associated with aortic valve stenosis, as a consequence of the Venturi effect in ventricular septal defects, or secondary to aortic disease including Marfans syndrome [3–6].

In patients with aortic regurgitation, the regurgitant blood flow increases left ventricular preload, potentially leading to dilation and eventual left ventricular failure over time. Most children with congenital aortic regurgitation are asymptomatic, with normal left ventricular dimensions and function, and may remain stable for several years before surgical repair may be needed [5]. In adults, well-established cut-off values exist for grading the severity of aortic regurgitation [7], however, corresponding cut-off values for pediatric patients are remain unclear [8].

This study investigated the prevalence of congenital aortic regurgitation in a large population-based cohort of newborns, described associated structural findings, and assessed for early signs of left ventricular volume overload.

## Materials and Methods

### Copenhagen Baby Heart Study

The Copenhagen Baby Heart Study is a population-based, prospective, multicenter study of newborns in Copenhagen, Denmark, focusing on cardiac structure and function. The study included 27,595 newborns, delivered in the three largest maternity wards in the Capital Region, between April 2016 and October 2018. The detailed methodology and cohort profile has previously been described [9, 10]. In short, all expectant parents from the catchment areas of the three hospitals were offered inclusion in the study. Information on parents, pregnancy, delivery, and newborn characteristics was collected through medical records. All included newborns were invited for an examination within 60 days after birth with a standardized transthoracic echocardiography, electrocardiogram, and pulse oximetry testing.

### Study Population and Aims

This study included all newborns with aortic regurgitation (both trivial and non-trivial) from the Copenhagen Baby Heart cohort. Newborns with non-trivial aortic regurgitation were matched 1:4 with newborns without aortic regurgitation from the same cohort (control group), based on sex, gestational age, birth weight, and age at time of examination.

The primary aim of this study was to determine the prevalence and severity of aortic regurgitation. Secondary objectives included assessing valve morphology, aortic dimensions, and left ventricular structure and function in newborns with non-trivial aortic regurgitation.

### Systematic Assessment of Aortic Regurgitation and Echocardiographic Analyses

A detailed description of the standardized transthoracic echocardiography protocol used in the Copenhagen Baby Heart Study has been published previously [9]. Views to detect aortic regurgitation included left parasternal long-axis and short-axis views and apical 5-chamber view with Color Doppler.

**Fig. 1:**
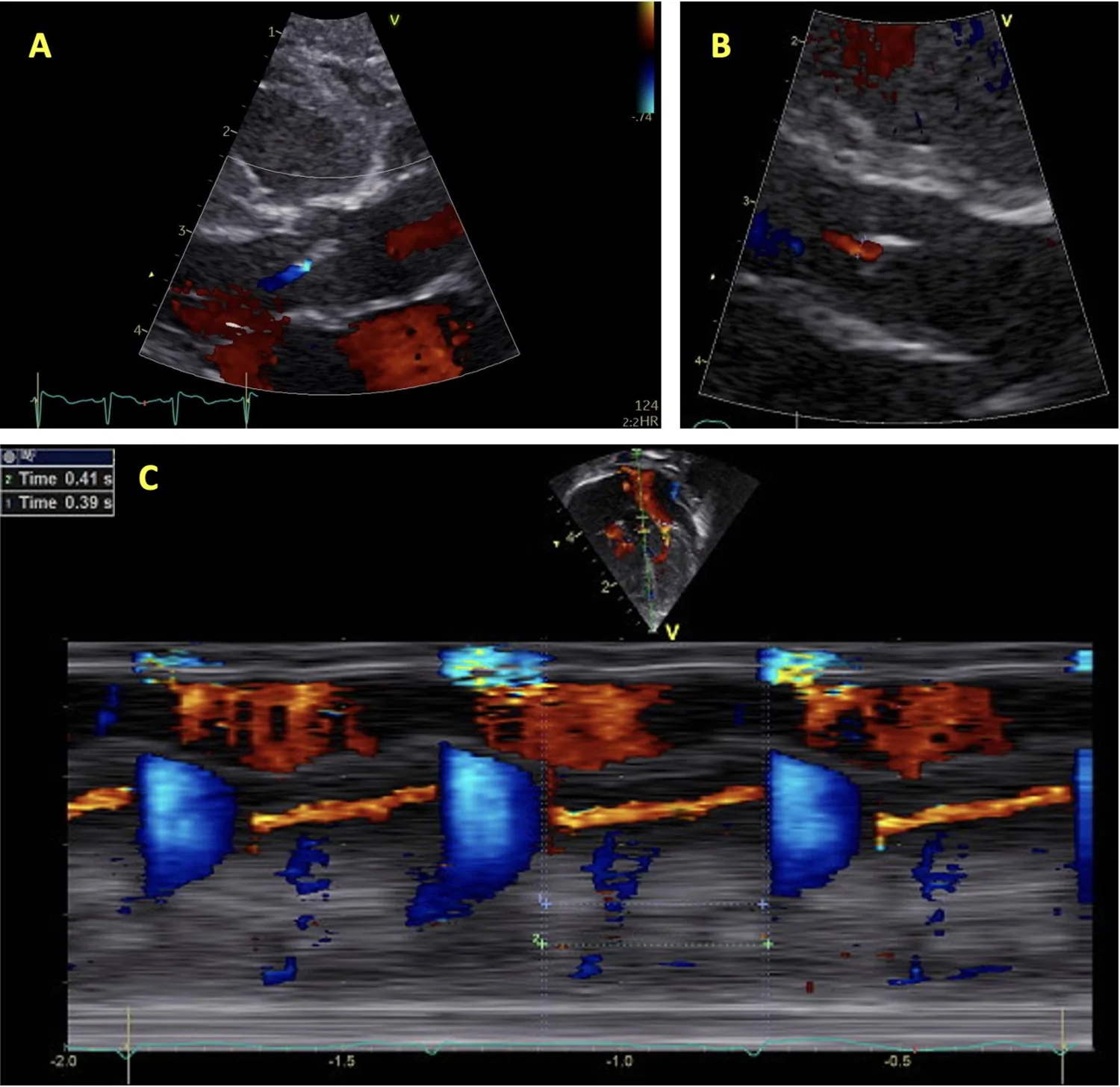
Echocardiographic examples of aortic regurgitation; **A** Presence of aortic valve regurgitation, seen in the parasternal long-axis view. **B** Measurement of the vena contracta aortic valve regurgitation in the parasternal long-axis view. **C** Measurement of the transvalvular regurgitant flow through the aortic valve in M-mode in the apical 5-chamber view

All transthoracic echocardiograms were systematically reviewed for aortic regurgitation by one of five echo-technicians. Aortic regurgitation was defined as a regurgitant flow across the aortic valve, visible in at least two out of three aforementioned views or in multiple cardiac cycles within a single view. If aortic regurgitation was suspected, the echocardiogram was reviewed by one of two physicians (A.F.F. and J.K.B.) to confirm or reject the diagnosis (no regurgitation). All confirmed cases was assessed qualitatively (A.F.F. and J.K.B.) as trivial or non-trivial aortic regurgitation. Trivial regurgitation was defined as a glimpse of visible regurgitant flow and/or a vena contracta diameter < 0.1 cm. Non-trivial regurgitation was defined as visual impression of more than trivial regurgitant flow, with severity graded based on vena contracta diameter [5, 8] measured in the parasternal long-axis view (Fig. 1B) in accordance with recommendations [8], or, when image quality was poor, in the apical 5-chamber view [5]. As echocardiographic cut-off values for aortic regurgitation severity are established only for adults [5, 8], and considering the differences in heart size between adults and newborns as well as the limited availability of neonatal echocardiographic normative data [9], we categorized non-trivial aortic regurgitation as mild (vena contracta ≥ 0.1 and < 0.2 cm), moderate (≥ 0.2 and < 0.3 cm), or severe (≥ 0.3 cm), using a modified version of the approach described by Zoghbi et al. [8].

Quantitative analyses of the structural and functional parameters of the left ventricle, aortic valve, and ascending aorta were performed in all newborns with non-trivial regurgitation and their matched controls, following published guidelines [11]. Transvalvular flow and diastolic duration were measured using Color Doppler and M-mode from the apical 5-chamber view (Fig. 1C) and presented as a ratio. The regurgitation/diastole ratio was included as a continuous parameter to explore potential associations between regurgitation burden and cardiac volumes and dimensions. Aortic diameter z-scores were calculated based on the Pediatric Heart Network echocardiographic reference formulas [12], with body surface area estimated using the Haycock formula [13]. All quantitative analyses were performed by one physician (A.F.F.), unblinded to aortic valve function.

### Interobserver Agreement

Interobserver variability and agreement were assessed using a blinded list of 40 randomly selected examinations, including cases with trivial, non-trivial, and no aortic regurgitation. The two reviewing physicians (A.F.F., J.K.B.) independently categorized each examination as trivial, non-trivial, or no aortic regurgitation. Agreement was assessed using Cohen’s kappa (κ) statistic for two observers, with κ > 0.80 indicating almost perfect agreement, κ = 0.61–0.80 substantial, κ = 0.41–0.60 moderate, κ = 0.21–0.40 fair, and κ < 0.20 poor agreement [14].

### Statistical Analyses

Categorical variables were presented as numbers and percentages, and continuous parameters as mean ± standard deviation (SD). Differences in continuous variables between newborns with non-trivial aortic regurgitation and the control group were assessed using independent t-tests, and differences in categorical variables were evaluated using Fischer’s exact test. Univariate linear regression was used to explore potential linear relationships between aortic regurgitation parameters (i.e., vena contracta and aortic regurgitation/diastole ratio) and left ventricular (end-systolic and end-diastolic volume, stroke volume) and aortic parameters (aortic root and ascending aorta diameter). The prevalence of aortic regurgitation was presented with a 95% confidence interval (CI) based on the Wilson score method without continuity correction [15]. Statistical significance was defined as *p* < 0.05. All statistical analyses were performed using R software v3.6.1.

## Results

###  Prevalence of Aortic Regurgitation and Subtypes

**Fig. 2:**
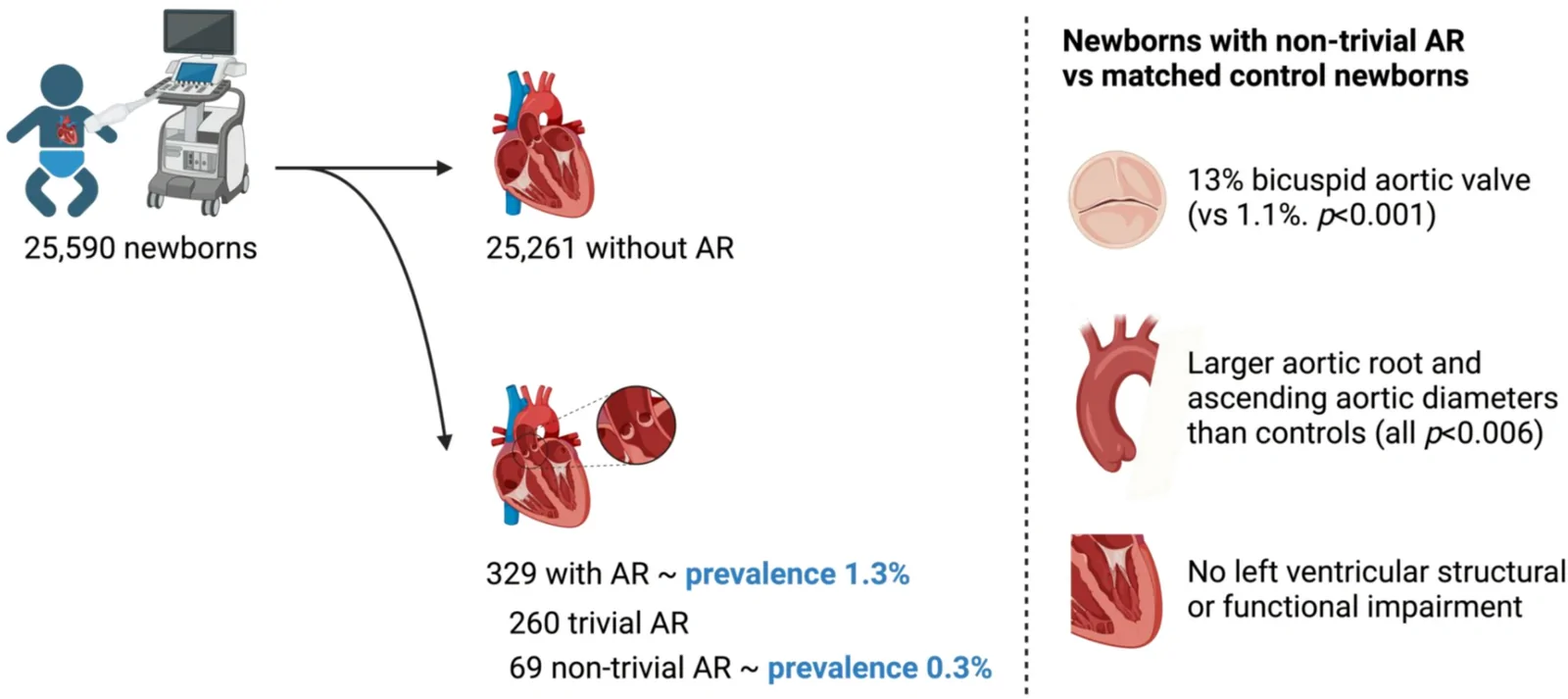
Overview of assessing the prevalence of congenital aortic regurgitation in a newborn cohort: This study aimed to determine the prevalence of aortic valve regurgitation in the Copenhagen Baby Heart Study Cohort – a contemporary neonatal cohort in Copenhagen, Denmark, in which 25,590 newborns were included and underwent transthoracic echocardiography. The study revealed an overall prevalence of aortic regurgitation of 1.3%, with non-trivial aortic regurgitation cases representing a 0.3% prevalence. Non-trivial cases were linked to larger aortic dimensions and a higher frequency of bicuspid valve (13.0%), but showed no signs of left ventricular structural or functional impairment. *AR* aortic regurgitation

A total of 25,590 newborns (52% males, median age 11 days) underwent transthoracic echocardiography in the Copenhagen Baby Heart Study. Aortic regurgitation was detected in 329 newborns, corresponding to a prevalence of 12.8 per 1,000 births (1.3%, 95% CI [1.15–1.43%]). Among these, 260 cases were classified as trivial aortic regurgitation, and 69 as non-trivial (2.7 per 1000 births, 0.3%, 95% CI [0.21–0.34%]). Of the 69 newborns with non-trivial aortic regurgitation, 54 were classified as mild, 15 as moderate, and none as severe (Fig. 2).

### Aortic Valve Morphology and Aortic Dimensions

Bicuspid aortic valve was more prevalent in newborns with non-trivial aortic regurgitation compared to controls (9 (13.0%) vs. 3 (1.1%), *p* < 0.001). Among newborns with a bicuspid aortic valve, one had moderate aortic regurgitation, while the remaining eight had mild aortic regurgitation. Newborns with non-trivial aortic regurgitation had significantly larger diameters of the aortic root and ascending aorta across all four measured segments compared to controls (*p* ≤ 0.006 for all; see Table. 2; Fig. 2).

**Table 1:** Maternal and offspring characteristics for newborns with non-trivial aortic valve regurgitation (AR), and matched controls (no AR) in the Copenhagen Baby Heart Study

Parameter	Non-trivial AR (Cases) *n* = 69	No AR (Controls) *n* = 276	*p*-value
*Newborn characteristics*			
Sex, female, n (%)	40 (58.0%)	161 (58.3%)	1
Gestational age, days	276.5 (11.3)	277.5 (10.0)	0.46
Age at examination, days	9.5 (7.4)	11.7 (7.0)	0.02
Birth length, cm	50.5 (6.0)	51.1 (3.4)	0.29
Birth weight, grams	3446.6 (532.8)	3413.6 (458.6)	0.61
APGAR score 5 min < 10, n (%)	4 (9.1%)	7 (3.6%)	0.25
*Maternal characteristics*			
Body Mass Index, kg/m^2^	22.9 (2.5)	23.1 (4.0)	0.74
Age, years	31.0 (4.7)	31.9 (4.4)	0.14

### Left Ventricular Structure and Function

Left ventricular volumes, wall thickness, and systolic function dit not differ significantly between newborns with non-trivial aortic regurgitation and controls, except for interventricular septum, which was slightly thinner in newborns with non-trivial aortic regurgitation (2.3 mm vs. 2.5 mm, *p* = 0.04) (Table 2).

**Table 2:** Comparison of left ventricular measures in newborns with non-trivial aortic valve regurgitation (AR), and no AR in newborns from the Copenhagen Baby Heart Study

Parameter	Non-trivial AR (Cases) (n=69)	No AR (Controls) (n=276)	*p*-value
*Left ventricle*			
Left ventricular end-diastolic diam., mm	19.8 (2.2)	19.6 (1.6)	0.47
Left ventricular end-systolic diam., mm	13.6 (2.0)	13.3 (1.3)	0.16
Left ventricular posterior wall diam. in end-diastole, mm	2.1 (0.6)	2.0 (0.5)	0.24
Interventricular septum diam. in end-diastole, mm	2.3 (0.4)	2.5 (0.5)	**0.04**
End diastolic volume, mL	12.7 (3.4)	12.3 (2.5)	0.27
End systolic volume, mL	4.8 (1.8)	4.5 (1.1)	0.13
Ejection fraction, %	62.4 (6.0)	63.5 (5.5)	0.19
Stroke volume, mL	7.8 (2.0)	7.8 (1.7)	0.90
Heart rate, bmp	132 (24.1)	143 (35.5)	**0.01**
Fractional shortening, %	31.6 (4.2)	32.4 (4.1)	0.20
Aortic valve and aortic root^*^			
Bicuspid aortic valve (BAV), n (%)	9 (13.0)	3 (1.1)	**<0.001**
Aortic valve annulus diam., mm	7.3 (0.7)	7.0 (0.7)	**0.005**
Aortic valve annulus diam., z-score	0.52 (1.25)	0.03 (1.13)	**0.006**
Sinuses of Valsalva diam., mm	10.3 (1.0)	9.7 (1.0)	**<0.001**
Sinuses of Valsalva diam., z-score	0.74 (1.43)	0.06 (1.23)	**<0.001**
Sinotubular junction diam., mm	8.4 (0.8)	8.0 (0.8)	**<0.001**
Sinotubular junction diam., z-score	0.66 (1.37)	0 (1.10)	**<0.001**
Ascending aorta diam., mm	10.0 (0.9)	9.4 (1.1)	**<0.001**
Ascending aorta diam., z-score	1.93 (1.33)	1.13 (1.31)	**<0.001**
*Other cardiac abnormalities*			
Other cardiac abnormalities, total n (%)**	22 (31.9)	93 (33.7)	0.89
Mitral valve regurgitation, n (%)	19 (27.5)	82 (29.7)	0.80
Barlow mitral valve disease, n (%)	0	1 (0.4%)	1
Ventricular septal defect, n (%)	3 (4.3)	16 (5.8)	0.78

*When excluding newborns with bicuspid aortic valve from the two groups, these dimensions were still significantly larger in the case vs. control group (*p* ≤0.001)

**Including mitral valve regurgitation, Barlow mitral valve disease, and ventricular septal defect. No cases of parachute mitral valve or pulmonary artery stenosis were seen in either groups

### Coexisting Congenital Heart Defects

The prevalence of other co-existing abnormalities (apart from bicuspid aortic valve), including mitral valve regurgitation of any severity, was similar between newborns with non-trivial aortic regurgitation and controls (31.9% vs. 33.7%, *p* = 0.89, Table 2).

### Interobserver Variability

The interobserver Cohen’s kappa coefficient for the detection of aortic regurgitation was κ = 0.55, indicating moderate agreement. For the classification of aortic regurgitation (trivial, non-trivial), the kappa coefficient was κ = 0.93, reflecting almost perfect agreement.

## Discussion

In this large, prospective, population-based study of over 25,000 newborns, aortic regurgitation was identified in 12.8 per 1000 newborns (1.3% prevalence), with non-trivial regurgitation found in 0.3%. Bicuspid aortic valve was 12 times more frequent in newborns with non-trivial aortic regurgitation compared to controls. Additionally, newborns with non-trivial aortic regurgitation had significantly larger aortic root and ascending aortic dimensions than controls, irrespective of valve morphology. These observations suggest congenital aortic regurgitation may involve intrinsic differences in aortic wall structure present from birth, potentially developed in fetal life, even without apparent valve malformation.

### Prevalence of Aortic Regurgitation

Literature on aortic regurgitation in newborns is scarce. Our findings surpass previously reported rates, except for an older review reporting aortic regurgitation in 0.3% of congenital heart defect cases (total *N* = 15,104, 1950–1973) at The Hospital of Sick Children, Toronto, Canada [16]. A systematic review and meta-analysis by Liu et al., encompassing 260 studies with over 130 million patients aged 0–6 years (1970–2017) of the global prevalence of congenital heart defects, reported a prevalence of aortic regurgitation of 0.02% (95% CI, 0.14–0.40%) based on 18 of the studies (1973–2013) [2]. Among more recent studies of infants, Iwashima et al. (2017) reported a prevalence of 0.09% (95% CI, 0.02–0.31%) in spontaneously conceived newborns (*n* = 2,336) and 0.25% (95% CI, 0.0004–0.014) in newborns after assisted reproduction (*n* = 401) based on echocardiography performed within four days of birth [17]. To the authors knowledge, no newer studies specifically addresss isolated congenital aortic regurgitation prevalence.

Our study, which included all severities of aortic regurgitation, found a prevalence of non-trivial aortic regurgitation three times higher than Iwashima’s [17]. This discrepancy might be explained by (1) the relatively small cohort size in Iwashima’s study, reflected in wider confidence intervals, (2) potential selection bias due to higher rates of congenital heart defects in assisted reproduction (including minor defects) [18], (3) improved detection of subtle valve insufficiencies with modern ultrasound technology, and (4) possible differences in cut-off values or thresholds for defining aortic regurgitation, which were not specified in Iwashima’s study.

Our prevalence estimates are based on systematic transthoracic echocardiography, in contrast to cases of aortic regurgitation detected due to symptoms, by auscultation, or as incidental findings. Discrepancies from Liu et al.’s overall findings [2] may stem from variations in study design (e.g. registry-based studies), differences in age groups, older ultrasound equipment, or the application or different extrapolation of adult aortic regurgitation thresholds (vena contracta < 0.3 cm as mild, > 0.6 cm as severe [3, 5]) [7], which were not addressed in Liu et al.’s analysis.

A recent retrospective study of 60 children (median age 14.8 years) with tricuspid aortic valves and trivial regurgitation reported no progression during a median follow-up of 2.1 years [19]. Further longitudinal studies are needed to elucidate the persistence and progression of congenital aortic regurgitation into later childhood and adolescence. Such data will be attainable through echocardiographic follow-up of the Copenhagen Baby Heart cohort.

### Coexisting Congenital Heart Defects and Aortic Dimensions

Pathology of the aortic valve leaflets, particularly bicuspid aortic valve, is a well-known cause of congenital aortic regurgitation [3, 6, 16], which aligns with our findings of significantly more cases of bicuspid aortic valve among newborns with non-trivial aortic regurgitation compared to controls, consistent with our previous findings of aortic regurgitation in 15% of newborns with bicuspid aortic valve versus 1.3% in controls [20].

Newborns with non-trivial aortic regurgitation exhibited slightly larger aortic dimensions than controls, with a linear relationship between the regurgitation/diastole ratio and aortic valve annulus diameter, suggesting decreased valve leaftet coaptation even with only slight increases in aortic dimensions. While aortic dilatation is common in newborns with bicuspid aortic valve [20], and has been reported in infants and children with significant aortic regurgitation [16], this study is the first to report subtle aortic enlargement in newborns with non-trivial aortic regurgitation, irrespective of valve morphology.

In newborns, congenital aortic regurgitation is often due to structural or functional abnormalities, such as bicuspid aortic valve, valve prolapse, or as a component in connective tissue disorders [16], or potentially represent a physiological phenomenon. In adults, aortic regurgitation can be caused by numerous etiologies, including congenital or acquired abnormalities of the aortic valve and/or aorta [4]. Recent research supports the notion that progressive aortic dilatation in adults with congenital heart defects is primary an intrinsic rather than secondary aortopathy [21]. Whether the slightly increased aortic dimensions observed in newborns with non-trivial aortic regurgitation indicate an underlying intrinsic aortic wall abnormality is unclear, however, it suggests that aortic enlargement linked to congenital aortic regurgitation commences already during fetal development. Our findings advocate a thorough examination of the aortic valve, root, and ascending aorta in newborns with aortic regurgitation. Longitudinal follow-up of the Copenhagen Baby Heart cohort will help identify those at risk of progression, potentially due to valvular pathology and/or intrinsic aortopathy, and distinguish these cases from physiological regurgitation with potential temporal variation, guiding appropriate counselling and follow-up [19, 22].

### Left Ventricular Function in Newborns with Aortic Regurgitation

In adults with chronic aortic regurgitation, increased left ventricular size and impaired function are strongly associated with adverse outcome [4]. In our study, left ventricular dimensions and function were similar between groups, except for a slightly thinner interventricular septum in newborns with non-trivial aortic regurgitation. While this finding could indicate early subtle changes, the previously reported poor reproducibility of wall thickness measurements in newborns [23] and the possibility of type I error must be considered. As all other left ventricular parameters were comparable, transthoracic echocardiography shortly after birth may be too early to detect secondary changes from aortic regurgitation. This aligns with the hypothesis that left ventricular functional impairment from aortic regurgitation is a result of long-term volume overload [4].

### Strengths and Limitations

The major strength of this study is the size and unselected nature of the cohort, with thorough and systematic echocardiographic examinations. Participants were included prenatally and parents were encouraged to participate regardless of prenatal findings. High-frequency probes and generally excellent imaging windows in newborns allow for optimal conditions and high-resolution echocardiography. However, echocardiography is observer dependent, and all quantitative measurements were performed by one physician (A.F.F.), which, while ensuring consistency, introduces a risk of systematic bias. The physician was not blinded to aortic valve function, introducing risk of bias in measurement outcomes. Nonetheless, neonatal echocardiographic image acquisition and measurements are generally reliable [23]. Interobserver agreement in this study was moderate for detection of aortic regurgitation, suggesting some very trivial cases may have been missed, but almost perfect agreement for distinguishing trivial from non-trivial aortic regurgitation, indicating that the prevalence of non-trivial aortic regurgitation was unlikely to be affected.

## Conclusion

We found the prevalence of aortic regurgitation to be 12.8 per 1000 newborns enrolled in a population study, with non-trivial aortic regurgitation in 2.7 per 1000. Newborns with non-trivial aortic regurgitation had larger aortic root and ascending aortic dimensions, irrespective of aortic valve morphology, and bicuspid aortic valve was frequent (13%). No early signs of left ventricular impairment was observed. Follow-up will help identify those at risk of progression, potentially due to valvular and/or aortic pathology, who may benefit from regular surveillance, and those who can be safely discontinued.

## Data Availability

Because of the sensitive nature of the data collected for this study, requests to access the dataset from qualified researchers trained in human subject confidentiality protocols may be sent to the Copenhagen Baby Heart Study (contact information available on baby-heart.dk).
